# Epigenetic Regulation Alters Biofilm Architecture and Composition in Multiple Clinical Isolates of Nontypeable Haemophilus influenzae

**DOI:** 10.1128/mBio.01682-18

**Published:** 2018-09-18

**Authors:** Kenneth L. Brockman, Patrick N. Azzari, M. Taylor Branstool, John M. Atack, Benjamin L. Schulz, Freda E.-C. Jen, Michael P. Jennings, Lauren O. Bakaletz

**Affiliations:** a Center for Microbial Pathogenesis, The Research Institute at Nationwide Children’s Hospital and The Ohio State University College of Medicine, Columbus, Ohio, USA; b Institute for Glycomics, Griffith University, Gold Coast, Queensland, Australia; c Australian Infectious Disease Research Centre, School of Chemistry and Molecular Biosciences, The University of Queensland, St. Lucia, Queensland, Australia; University of Washington

**Keywords:** DNABII, HU, NTHI, eDNA, otitis media, phasevarion

## Abstract

Biofilms play a critical role in the colonization, persistence, and pathogenesis of many human pathogens. Multiple mucosa-associated pathogens have evolved a mechanism of rapid adaptation, termed the phasevarion, which facilitates a coordinated regulation of numerous genes throughout the bacterial genome. This epigenetic regulation occurs via phase variation of a DNA methyltransferase, Mod. The phasevarion of nontypeable Haemophilus influenzae (NTHI) significantly affects the severity of experimental otitis media and regulates several disease-related processes. However, the role of the NTHI phasevarion in biofilm formation is unclear. The present study shows that the phasevarions of multiple NTHI clinical isolates regulate *in vitro* biofilm formation under disease-specific microenvironmental conditions. The impact of phasevarion regulation was greatest under alkaline conditions that mimic those known to occur in the middle ear during disease. Under alkaline conditions, NTHI strains that express the ModA2 methyltransferase formed biofilms with significantly greater biomass and less distinct architecture than those formed by a ModA2-deficient population. The biofilms formed by NTHI strains that express ModA2 also contained less extracellular DNA (eDNA) and significantly less extracellular HU, a DNABII DNA-binding protein critical for biofilm structural stability. Stable biofilm structure is critical for bacterial pathogenesis and persistence in multiple experimental models of disease. These results identify a role for the phasevarion in regulation of biofilm formation, a process integral to the chronic nature of many infections. Understanding the role of the phasevarion in biofilm formation is critical to the development of prevention and treatment strategies for these chronic diseases.

## Abstract (precis)

Upper respiratory tract infections are the number one reason for a child to visit the emergency department, and otitis media (middle ear infection) ranks third overall. Biofilms contribute significantly to the chronic nature of bacterial respiratory tract infections, including otitis media, and make these diseases particularly difficult to treat. Several mucosa-associated human pathogens utilize a mechanism of rapid adaptation termed the phasevarion, or phase variable regulon, to resist environmental and host immune pressures. In this study, we assessed the role of the phasevarion in regulation of biofilm formation by nontypeable Haemophilus influenzae (NTHI), which causes numerous respiratory tract diseases. We found that the NTHI phasevarion regulates biofilm structure and critical biofilm matrix components under disease-specific conditions. The findings of this work could be significant in the design of improved strategies against NTHI infections, as well as diseases due to other pathogens that utilize a phasevarion.

## INTRODUCTION

Nontypeable Haemophilus influenzae (NTHI) naturally resides asymptomatically within the human nasopharynx (NP). However, changes in NTHI or the host can result in increased virulence and subsequent disease. NTHI causes multiple human respiratory tract diseases, which include otitis media (OM), sinusitis, conjunctivitis, and pneumonia (1). NTHI is the predominant pathogen in the establishment of chronic and recurrent OM (2), diseases that result in conductive hearing loss and often require costly surgical intervention (3–5).

To date, phasevarions have been identified in Haemophilus influenzae, Streptococcus pneumoniae, Moraxella catarrhalis, Helicobacter pylori, Kingella kingae, and Neisseria species (6–11). The phasevarion mechanism simultaneously regulates the expression of multiple genes throughout the bacterial chromosome via phase variation of the DNA methyltransferase, Mod. Expression of Mod results in site-specific chromosomal methylation, which alters the expression of genes within the regulon. This epigenetic regulatory mechanism results in two genetically identical, yet phenotypically distinct, subpopulations of bacteria referred to as “*mod* OFF” when the Mod protein is not expressed and “*mod* ON” when the Mod protein is expressed. The phasevarions of multiple species have been shown to regulate disease-related phenotypes *in vitro* and virulence in experimental models of disease. In NTHI, ModA has been identified as the phase variable methyltransferase, of which over 21 distinct alleles have been identified. However, two-thirds of NTHI strains isolated from the NP or middle ear (ME) of children with chronic and/or recurrent OM have one of just five alleles (*modA2*, -*4*, -*5*, -*9*, or -*10*) (12). Phasevarions controlled by these five predominant alleles regulate expression of virulence determinants, which include potential vaccine antigens. ModA2 of NTHI strain 723 has also been shown to significantly impact disease severity in a chinchilla model of OM (13).

During the course of OM, NTHI cells in the NP ascend the eustachian tube and gain access into the ME, initially resulting in acute otitis media (AOM) that can later evolve to chronic and/or recurrent OM. The microenvironments of the ME, sinuses, and lungs are very different from those of the NP and one another. In healthy individuals, the NP is at 34°C, and association with the mucosal membranes provides bacteria with nutrients and a pH neutral surface (14). In contrast, the ME has varied nutrient availability and is warmer at 37°C, and ME effusions increase in pH as disease progresses (15–17). It has been demonstrated that Haemophilus influenzae responds to changes in pH by regulating mechanisms of pH homeostasis or induction of biofilm formation in a strain-dependent manner (18). The formation of a biofilm contributes significantly to the chronic and recurrent nature of diseases, such as OM.

Bacteria within biofilms are more resistant to environmental stresses, antimicrobial treatments, and host immunity due to multiple factors. The protective nature of the extracellular polymeric substance (EPS), or biofilm matrix, provides structural integrity to the biofilm and acts a physical barrier to separate cells within the biofilm from the extracellular milieu (19–23). Biofilm architecture and structure play a key role in the exchange of nutrients and waste within the biofilm (24, 25). Metabolic quiescence, induced by the stringent response, and changes in the growth rate of bacteria within a biofilm significantly contribute to increased resistance to antibiotics. A shift in bacterial metabolism within Pseudomonas biofilms, due to nutrient or oxygen limitation, has been shown to confer increased resistance to antibiotics (26, 27). NTHI biofilms are known to be more resistant to antibiotic killing than planktonic bacteria, yet the mechanism behind this observation is not fully understood. Proteomic analysis by Post et al. revealed decreased energy metabolism and protein synthesis by NTHI within biofilms compared to planktonic NTHI (28), which may contribute to increased antibiotic resistance.

The NTHI phasevarions potentially regulate numerous genes and gene products involved in multiple aspects of biofilm formation. Here we assess the role of the five most predominant *modA* alleles in biofilm formation under conditions specifically designed to mimic the microenvironments of the human nasopharynx, middle ear, and middle ear during disease.

## RESULTS

### Multiple ModA phasevarions regulate biofilm formation under diverse environmental conditions.

**FIG 1:**
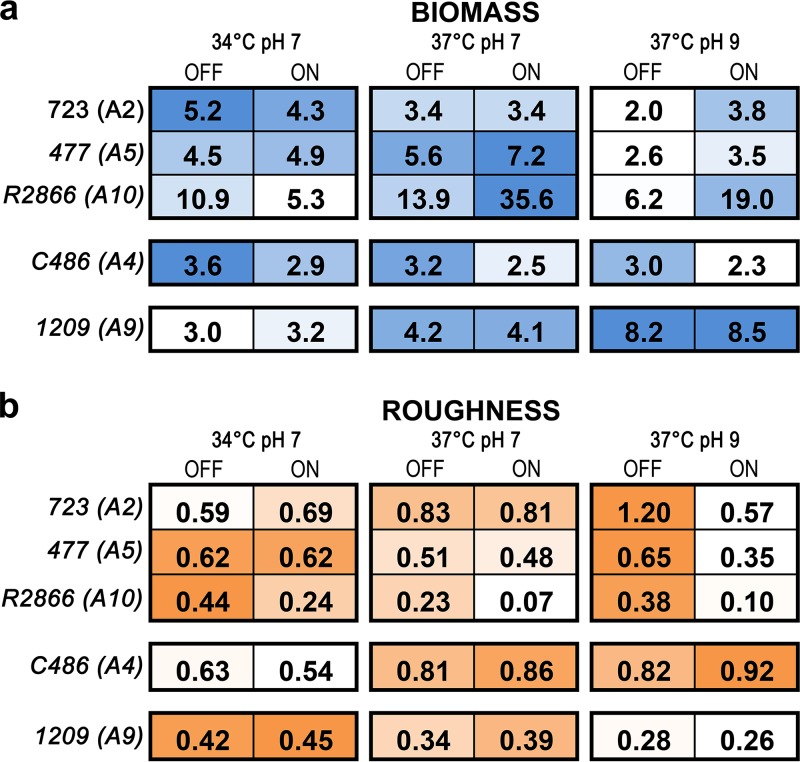
Biomass and roughness of biofilms formed by NTHI clinical isolates that represent the 5 most common *modA* alleles. (a and b) Biofilms were formed by NTHI clinical isolates that represent the most common *modA* alleles: strain 723 (*modA2*), strain 477 (*modA5*), strain R2866 (*modA10*), strain C486 (*modA4*), and strain 1209 (*modA9*). Biofilms were grown for 24 h under conditions that mimic the human NP (34°C, pH 7), the healthy ME (37°C, pH 7), or the ME during chronic disease (37°C, pH 9). COMSTAT2 analysis was performed to determine (a) biomass and (b) roughness. Individual values are colored to indicate biomass (blue) or roughness (orange) for each strain. Darker colors indicate greater values. Strains were grouped based on biofilm formation at 37°C, pH 9. Values are an average of duplicate biofilms from 3 independent experiments.

Representative strain pairs (*modA* ON and *modA* OFF) for each of the 5 most common alleles, *modA2*, -*4*, -*5*, -*9*, and -*10*, were tested for their ability to form biofilms within the wells of a chambered cover glass. NTHI strains 723, C486, 477, and 1209 were all isolated from the middle ears of children with otitis media. Strain R2866 is a blood isolate collected from a child with bacterial meningitis. For all of the strains tested in Fig. 1, the *modA* allele was free to phase vary or switch, but populations were predominantly (>90%) in the indicated *modA* status at the time of inoculation. Biofilms were formed at 34°C and a neutral pH (pH 7), 37°C with a neutral pH (pH 7), or 37°C with an alkaline pH (pH 9). Biofilms were started in brain heart infusion supplemented with hemin and NAD (sBHI) buffered to the indicated pH (7 or 9). Due to factors such as acidifying metabolic by-products, the pH of sBHI adjusted to a pH of 9 dropped and stabilized near a pH of 8 (see Fig. S1 in the supplemental material). As the pH of middle ear fluids collected from children with OM ranges from pH 8 to 9, we selected medium with an initial pH of 9 (16). The heat map in Fig. 1 represents the average biomass (μm^3^/μm^2^) and roughness (*R_a_*) of biofilms formed by each strain under these three representative conditions. ModA status significantly affected biofilm formation by 3 of the 5 strains (Fig. 1; see Table S1 in the supplemental material). Under conditions that mimic those of the ME with chronic effusions (37°C, pH 9), NTHI strains 723 (*modA2*), 477 (*modA5*), and R2866 (*modA10*) produced biofilms that were larger in biomass and thickness and significantly less rough when ModA was expressed (ON status) compared to when ModA was not expressed (OFF status) (Fig. 1, *P  < *0.01; Table S1). Biofilm roughness is a measure of structural heterogeneity, and increased roughness indicates more variation in the biofilm surface. ModA expression did not affect biofilm formation by NTHI strain C486 (*modA4*) or NTHI strain 1209 (*modA9*), with no differences observed between variants in either the ON or OFF status under the conditions tested; however, alkaline conditions did significantly affect biofilm formation by strain 1209 irrespective of *modA9* status (Fig. 1; α < 0.01; Table S1). These results indicated that phasevarion status significantly affects biofilm formation by multiple NTHI clinical isolates and that microenvironmental changes significantly impact how NTHI strains form biofilms in both phasevarion-dependent and -independent manners.

10.1128/mBio.01682-18.1 FIG S1 Effect of NTHI growth on the pH of buffered sBHI. NTHI cells were inoculated into sBHI supplemented with 0.1 M HEPES or 0.1 M TAPS and adjusted to a pH of 6, 7, 8, or 9. Medium was collected after 6 or 12 h, and the pH was measured. sBHI buffered to a starting pH of 9 dropped to an approximate pH of 8. sBHI buffered to a starting pH of 8 dropped to an approximate pH of 7. sBHI buffered to a starting pH of 7 dropped to an approximate pH of 6.4. sBHI buffered to a starting pH of 6 dropped to an approximate pH of 5.2. Starting pHs of 7 and 9 were selected to represent neutral and alkaline conditions, respectively. Download FIG S1, TIF file, 0.1 MB. Copyright © 2018 Brockman et al. 2018 Brockman et al. This is an open-access article distributed under the terms of the Creative Commons Attribution 4.0 International license.

10.1128/mBio.01682-18.3 TABLE S1 Statistical analysis of biomass and roughness of biofilms formed by NTHI clinical isolates that represent the 5 most common *modA* alleles. Student’s *t* test was performed to compare *modA* OFF versus *modA* ON populations under each of the conditions tested, as indicated. One-way ANOVA with multiple comparisons (Holms-Sidak) was used to compare the effect of microenvironmental conditions among members of a single *modA* subpopulation. Download Table S1, XLSX file, 0.1 MB. Copyright © 2018 Brockman et al. 2018 Brockman et al. This is an open-access article distributed under the terms of the Creative Commons Attribution 4.0 International license.

### ModA2 regulates biofilm structure at alkaline pH.

**FIG 2:**
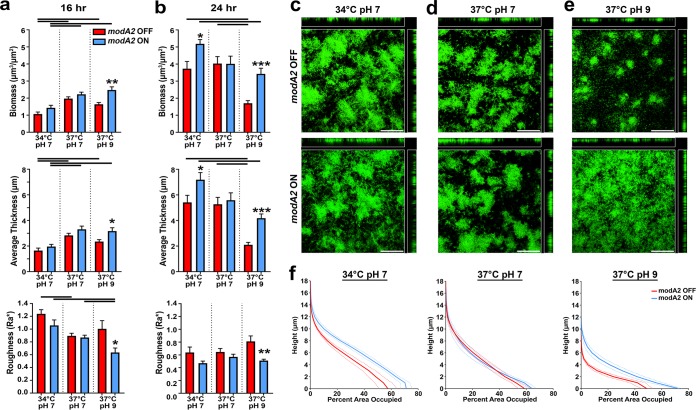
Biofilm formation by NTHI strain 723. (a and b) Biomass, average thickness, and roughness of *modA2* locked ON and *modA2* locked OFF biofilms grown for (a) 16 h or (b) 24 h. Biofilms were analyzed by COMSTAT2, and values are shown as mean ± standard error of the mean. Lines over graphs indicate significance due to growth condition by one-way analysis of variance (ANOVA) with Tukey’s multiple comparisons (α < 0.05). Asterisks denote significance between subpopulations under each condition: *, *P*  < 0.05, **, *P*  < 0.01, and ***, *P*  < 0.001, by Student's *t* test. (c to e) Representative orthogonal image renderings of *modA2* locked ON and *modA2* locked OFF biofilms grown at (c) 34°C at pH 7, (d) 37°C at pH 7, and (e) 37°C at pH 9. Scale bars, 100 μm. (f) Average percentage of area occupied by bacteria at each 1-μm optical section (“layer”). Dashed lines indicate standard error of the mean.

To identify key phasevarion-regulated factors that contributed to the observed differences in biofilm structure, we first investigated biofilm formation by NTHI strain 723. NTHI strain 723 carries the *modA2* allele and has been studied *in vitro* as well as in the chinchilla model of OM (12, 13, 29). Locked versions of NTHI strain 723, in which *modA2* is unable to phase vary, were utilized, and biofilm formation was assessed after both 16 and 24 h (29). As was observed with the enriched populations that were free to phase vary (Fig. 1), biofilms formed for 16 or 24 h by strain 723 *modA2* locked ON and *modA2* locked OFF were of similar total biomasses at 37°C and a neutral pH (Fig. 2a and b). In contrast, biofilms formed for 16 or 24 h by *modA2* locked ON at an alkaline pH had significantly greater biomass than those formed by *modA2* locked OFF (Fig. 2a and b; *P  < *0.001). Additionally, biofilms formed by *modA2* locked ON bacteria and *modA2* locked OFF bacteria exhibited major differences in biofilm architecture and bacterial distribution under alkaline conditions (Fig. 2e). At neutral pH, regardless of *modA2* status, biofilms had dense tower-like structures (viewed as green) with interspersed water channels (viewed as black), characteristic of biofilms formed by other NTHI strains under standard laboratory conditions (Fig. 2c and d) (30, 31). *modA2* locked OFF bacteria formed biofilms with similar tower-like structures under alkaline conditions, but with significantly less biomass than those formed at a neutral pH (Fig. 2b and e; *P  < *0.005). In contrast, *modA2* locked ON bacteria formed biofilms with a distinctive architecture that lacked tower-like structures and formed a mat-like biofilm that was significantly denser at the base of the biofilm compared to *modA2* OFF biofilms (compare Fig. 2e top and bottom images; *P  < *0.01, unpaired *t* test). To confirm that these observations were not due to differences in growth rate, planktonic growth in sBHI buffered to pH 6, 7, 8, or 9 was assessed. There were no differences in growth rates between the ON and OFF subpopulations at any of the pHs tested (see Fig. S2 in the supplemental material).

10.1128/mBio.01682-18.2 FIG S2 Growth of NTHI strain 723 *modA2* ON and *modA2* OFF subpopulations. Bacteria were diluted into buffered sBHI adjusted to a starting pH of 6, 7, 8, or 9 and seeded into wells of a 96-well flat-bottom plate. Plates were incubated shaking at either 34 or 37°C with 5% atmospheric CO_2_. Absorbance at 490 nm was recorded every 15 min for 12 h. No significant differences in planktonic growth rates occurred between the *modA2* ON (circles) or OFF (squares) populations at any of the temperatures or pHs tested. Download FIG S2, TIF file, 0.5 MB. Copyright © 2018 Brockman et al. 2018 Brockman et al. This is an open-access article distributed under the terms of the Creative Commons Attribution 4.0 International license.

In order to compare these architectural differences in greater detail, we determined the area occupied by layer for each population. The area occupied by layer is a calculation of the amount (or percentage) of bacterial biomass that is present within each 1-μm optical section of the biofilm taken from the base of the biofilm to the top. These data were plotted wherein the layer closest to the glass surface is at the bottom of the *y* axis and the top of the biofilm (farthest from the surface) is at the top of the *y* axis. At 34°C at pH 7, *modA2* locked ON biofilms were denser near the base of the biofilm compared to those formed by *modA2* locked OFF bacteria, but were of similar densities and heights near the top of the biofilm (Fig. 2f). There were no significant differences between biofilms grown at 37°C at pH 7. Under alkaline conditions, *modA2* locked ON biofilms were composed of significantly greater biomass throughout the biofilm compared to *modA2* locked OFF biofilms. *modA2* locked ON biofilms were significantly denser at the bottom of the biofilm and occupied up to 75% of the glass surface compared to 50% by *modA2* locked OFF biofilms (Fig. 2f; *P*  < 0.05).

**FIG 3:**
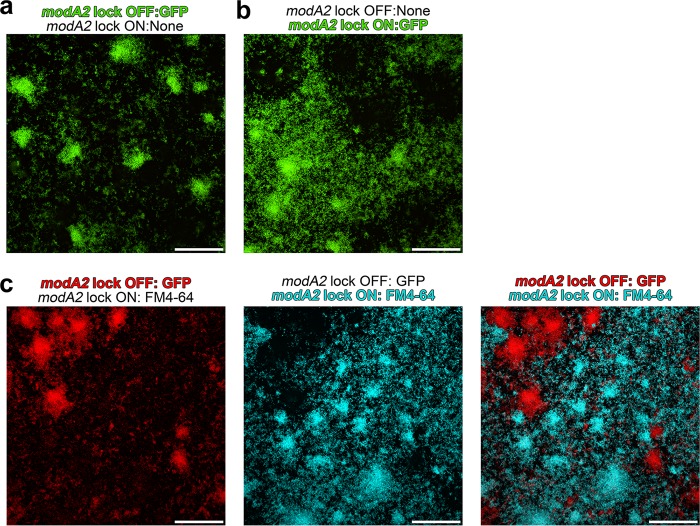
Distribution of individual subpopulations within an NTHI strain 723 mixed biofilm. Biofilms were formed by a 1:1 mixture of NTHI strain 723 *modA2* locked OFF and *modA2* locked ON bacteria. Biofilm cultures were incubated for 24 h at 37°C at pH 9. (a) Representative biofilm formed by a 1:1 mixture of *modA2* locked OFF bacteria that constitutively expresses GFP and nonfluorescent *modA2* locked ON. The GFP fluorescent *modA2* OFF population formed dense tower-like structures, shown in green. (b) Representative biofilm formed by a 1:1 mixture of nonfluorescent *modA2* locked OFF bacteria and *modA2* locked ON bacteria that constitutively expresses GFP. The GFP fluorescent *modA2* ON population formed a more mat-like biofilm, shown in green. (c) Representative biofilm formed by a 1:1 mixture of *modA2* locked OFF bacteria that constitutively expresses GFP and nonfluorescent *modA2* locked ON bacteria. Biofilms were counterstained with the bacterial outer membrane stain FM4-64 to visualize the *modA2* ON population, which does not express GFP. *modA2* locked OFF (pseudocolored red) is shown in the left panel, and *modA2* locked ON (pseudocolored blue) is shown in the middle panel. The localization and distribution of both subpopulations are shown as a merged image in the panel on the right. Scale bars, 100 μm.

In nature, NTHI strains naturally exist as a mixture of both *modA2* ON and *modA2* OFF subpopulations. To verify that subpopulations maintained their unique biofilm phenotype when grown together, biofilms were formed with an equal 1:1 mixture of *modA2* locked OFF bacteria and *modA2* locked ON bacteria, in which the *modA2* locked OFF or *modA2* locked ON population had been engineered to constitutively express green fluorescent protein (GFP). The bacteria were incubated at 37°C at pH 9 to mimic the conditions of the ME during disease, and biofilms were allowed to form for 24 h. Each subpopulation maintained its distinct phenotype in which *modA2* locked OFF bacteria formed discrete tower-like structures (Fig. 3a) and *modA2* locked ON bacteria formed a more mat-like biofilm (Fig. 3b). When mixed biofilms were counterstained with the membrane stain FM4-64, to visualize the nonfluorescent bacteria, a mat-like biofilm formed by *modA2* locked ON bacteria (pseudocolored blue) surrounded dense tower-like structures formed by *modA2* locked OFF bacteria (pseudocolored red) (Fig. 3c). Taken together, these data suggested that the ModA2 phasevarion regulates biofilm formation under alkaline conditions, such as those in the ME during chronic disease, with both *modA2* ON and *modA2* OFF subpopulations contributing substantially to the large biofilms we have previously observed formed by populations that contain a mixture of *modA2* ON bacteria and *modA2* OFF bacteria (13). As disease progresses, selection for a predominant *modA* status, such as the shift to *modA2* ON status within the chinchilla middle ear (12), will influence the resultant character of the biofilm within each specific niche.

### ModA5 regulates biofilm formation in response to alkaline conditions.

**FIG 4:**
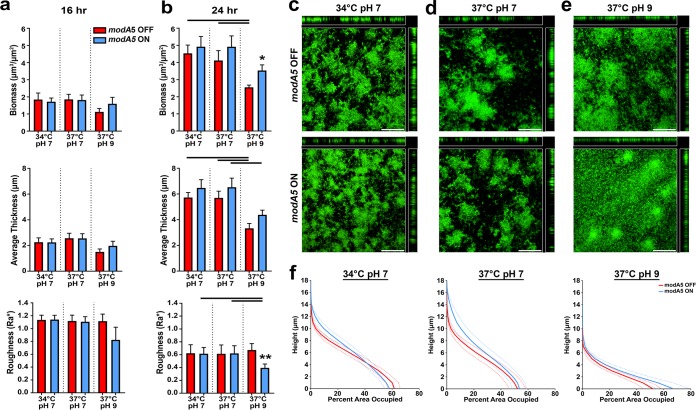
Biofilm formation by NTHI strain 477. (a and b) Biomass and average thickness and roughness of *modA5* ON and *modA5* OFF biofilms grown for (a) 16 h or (b) 24 h. Biofilms were analyzed by COMSTAT2, and values are shown as mean ± standard error of the mean. Lines over graphs indicate significance due to growth condition by one-way ANOVA with Tukey’s multiple comparisons (α < 0.05). Asterisks denote significance between subpopulations under each condition: *, *P*  < 0.01, and **, *P*  < 0.001, Student's *t* test. (c to e) Representative orthogonal image renderings of *modA5* ON and *modA5* OFF biofilms grown at (c) 34°C at pH 7, (d) 37°C at pH 7, and (e) 37°C at pH 9. Scale bars, 100 μm. (f) Average percentage of area occupied by bacteria at each individual 1-μm optical section (“layer”). Dashed lines indicate standard error of the mean.

To determine if the above observations were restricted to strains that carry the *modA2* allele, we selected a second clinical isolate, NTHI strain 477, to assess biofilm formation by a strain that contained a different *modA* allele than NTHI strain 723. NTHI strain 477 carries a *modA5* allele that is free to phase vary or switch *modA* status. ModA5 recognizes and methylates a different DNA sequence than other ModA methyltransferases and thus regulates a unique set of genes (12). Biofilm formation was studied with *modA5* ON and *modA5* OFF populations that were greater than 90% ON or OFF at time of inoculation, but free to switch (12). As such, biofilm formation by strain 477 was expected to be more heterogeneous than those formed by pure strain 723 locked populations that were unable to phase vary. After 16 h of biofilm growth, there were no significant differences between the biofilms as assessed by COMSTAT (Fig. 4a). However, after 24 h there were significant differences in biofilm formation between the *modA5* subpopulations when grown at 37°C at pH 9, like what was observed with strain 723 (compare Fig. 4b and Fig. 2b). At an alkaline pH, *modA5* ON bacteria formed biofilms that were more mat-like in architecture with significantly greater biomass than those formed by *modA5* OFF bacteria (Fig. 4b and e; *P*  = 0.01, unpaired *t* test). *modA5* ON bacteria also formed biofilms with greater biomass at the base of the biofilm, compared to *modA5* OFF biofilms; these data are presented as area occupied by layer (Fig. 4f). At a neutral pH, both strain 477 subpopulations formed biofilms with towers and intervening water channels, as was observed with strain 723 at a neutral pH (Fig. 2c and d). To ensure that the biofilm phenotypes observed were due to the indicated *modA5* status and not switching, we assayed for this and found that the relative *modA5* status for each of the populations (>90% *modA5* ON or >90% *modA5* OFF at time of inoculation) had not significantly shifted after 24 h of biofilm formation under any condition tested (see Table S2 in the supplemental material). Thereby, even when *modA5* was free to phase vary, biofilm formation was significantly different between *modA5* ON and *modA5* OFF subpopulations when grown at an alkaline pH.

10.1128/mBio.01682-18.4 TABLE S2 Ratio of *modA5* OFF to *modA5* ON in 24-h biofilms. Biofilms were formed by NTHI strain 477 populations in the predominantly (>90%) *modA5* OFF or *modA5* ON status at time of seed (inoculation into well of chambered coverglass). Planktonic bacteria as well as adherent biofilm bacteria were collected after 24 h at 34°C at pH 7, 37°C at pH 7, or 37°C at pH 9. Fragment length analysis was performed as described previously (12) to identify any shift in population status. No significant shift was observed for any of the samples or conditions tested. Download Table S2, XLSX file, 0.1 MB. Copyright © 2018 Brockman et al. 2018 Brockman et al. This is an open-access article distributed under the terms of the Creative Commons Attribution 4.0 International license.

### The ModA2 phasevarion regulates proteins required for biofilm formation and structural stability.

**FIG 5:**
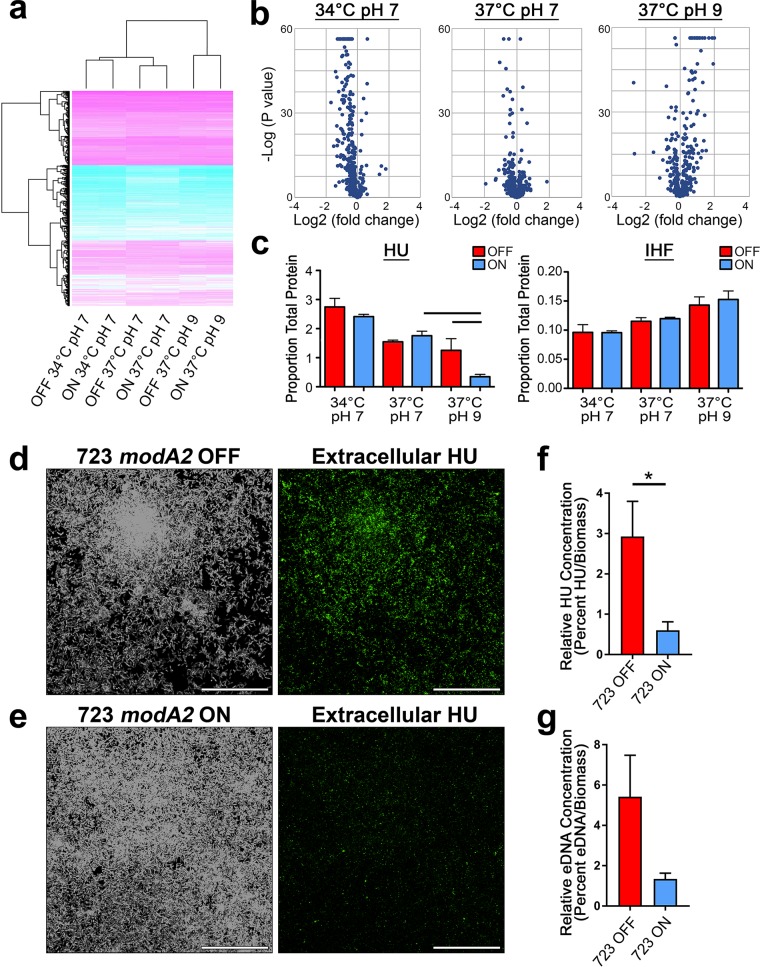
Relative protein abundance within NTHI strain 723 biofilms. (a) Clustered heat map of protein abundance within biofilms formed by NTHI strain 723 at 34°C at pH 7, 37°C at pH 7, or 37°C at pH 9. (b) Volcano plots of protein abundance between *modA2* locked ON biofilms versus *modA2* locked OFF biofilms. (c) Relative protein abundance of the DNABII proteins HU and IHF as percentage of proportion of total protein. Lines over the graph indicate adjusted *P* value of <1.00E−12. (d to e) Laser-scanning confocal images of (d) *modA2* locked OFF biofilms and (e) *modA2* locked ON biofilms imaged for total biomass (left [white]) and extracellular HU (right [green]). Scale bars, 30 μm. (f) Relative HU abundance within the biofilms normalized to total biomass. *, *P*  < 0.05, unpaired *t* test. (g) Relative eDNA abundance within the biofilms normalized to total biomass.

To now elucidate specific differences between the proteomes of biofilms formed under alkaline conditions, proteomic analysis was performed to determine relative protein abundances within biofilms formed by each *modA2* subpopulation. Sequential window acquisition of all theoretical mass spectra (SWATH-MS) (32), which is a data-independent acquisition (DIA) mass spectrometry (MS) approach, was used to determine relative quantification of every detectable peptide within the biofilm sample. Biofilms were formed by NTHI strain 723 *modA2* locked populations for 16 h under conditions designed to mimic the microenvironments of the human nasopharynx (34°C, pH 7), healthy middle ear (37°C, pH 7), or middle ear during chronic OM (37°C, pH 9). The 16-h time point was selected because significant differences in biofilm structure were observed (Fig. 2) and to avoid potential effects of replacing the growth medium. The biofilms were rinsed to remove any nonadherent bacteria, and then the entire biofilm mass was collected in order to analyze the total protein content of the biofilm; this included total bacterial proteins as well as proteins integrated into the biofilm extracellular matrix. SWATH-MS proteomic analysis identified the abundance of a total of 644 proteins in each sample. The most important environmental determinant of protein expression in these samples was pH, followed by temperature, as depicted in the clustered heat map in Fig. 5a. Significant differences in protein abundance between the *modA2* locked ON and *modA2* locked OFF populations at each condition are represented as volcano plots (Fig. 5b). The *x* axis indicates relative fold difference in protein abundance in *modA2* ON biofilms compared to *modA2* OFF biofilms; the *y* axis indicates statistical significance. The greatest differences in ModA2-dependent protein expression occurred at 37°C at pH 9. Proteins with significant changes in abundance (log_2_ fold change [FC] of ≥1 or ≤−1, *P  < *0.05) between *modA2* locked ON biofilms and *modA2* locked OFF biofilms are presented in Table S3 in the supplemental material.

10.1128/mBio.01682-18.5 TABLE S3 NTHI strain 723 SWATH results. Relative protein abundance in biofilms formed by NTHI strain 723 *modA2* locked ON and *modA2* locked OFF populations at 34°C at pH 7, 37°C at pH 7, or 37°C at pH 9. Log_2_ fold change of ≥1. Download Table S3, XLSX file, 0.1 MB. Copyright © 2018 Brockman et al. 2018 Brockman et al. This is an open-access article distributed under the terms of the Creative Commons Attribution 4.0 International license.

At 34°C at pH 7, we observed a 2-fold or greater reduction (log_2_ FC of ≤−1) in relative abundance of 15 proteins within biofilms formed by *modA2* locked OFF bacteria compared to those formed by *modA2* locked ON bacteria. The abundance of only one protein changed greater than 2-fold at 37°C at pH 7. This protein, an alkylhydroperoxidase (723_00583), was more abundant in *modA2* locked ON biofilms compared to *modA2* locked OFF biofilms (log_2_ FC of −1.05). Under conditions that mimic those within the ME during disease (37°C, pH 9), there was a 6.6-fold increase (log_2_ FC of −2.72) of a putative Fe^2+^-trafficking protein (723_00852) in the *modA2* locked ON biofilms compared to the *modA2* locked OFF biofilms. A total of 19 proteins were 2-fold or less (log_2_ FC of ≤−1) abundant in the *modA2* locked ON biofilms, and 5 of these proteins were ribosomal proteins (Table S3). There was also a significant decrease in abundance of the DNABII DNA-binding protein HU (723_01259) within biofilms formed by *modA2* locked ON bacteria compared to those formed by *modA2* locked OFF bacteria (log_2_ FC = 1.70, *P  < *1E−15). HU abundance was only significantly decreased within biofilms formed by *modA2* locked ON bacteria at pH 9, the same conditions under which the *modA2* locked ON subpopulation formed the distinct mat-like biofilm (Fig. 5c). There were no significant differences in the abundance of the DNABII DNA-binding protein IHF (723_00064) within these early biofilms (Fig. 5c). These results indicated the role of the phasevarion in regulation of numerous proteins, but more intriguing was the fact that the greatest changes in protein abundance occurred under physiologically relevant conditions, such as the pHs and temperatures that mimic those within the nasopharynx and diseased middle ear.

To confirm the decreased abundance of HU within biofilms formed by *modA2* locked ON bacteria at pH 9, we performed immunofluorescence. Biofilms were formed by the *modA2* locked OFF and *modA2* locked ON subpopulations for 16 h at 37°C at pH 9 and then labeled with a polyclonal antibody directed against NTHI HU in order to determine the relative abundance and distribution of extracellular HU within these biofilms. Significantly more relative HU was present within biofilms formed by *modA2* locked OFF bacteria than in biofilms formed by *modA2* locked ON bacteria (compare Fig. 5d and e and see Fig. 5f; *P*  = 0.03, unpaired *t* test) confirming the SWATH-MS results.

HU binds to DNA within the extracellular matrix of biofilms. Therefore, we utilized immunofluorescence to determine potential differences in extracellular DNA (eDNA) within biofilms formed under alkaline conditions (37°C, pH 9). Sixteen-hour biofilms formed by the 723 *modA2* locked OFF or 723 *modA2* locked ON populations were labeled for the presence of eDNA with commercial antibody directed against double-stranded DNA (dsDNA). Biofilms formed by the *modA2* OFF population contained more relative eDNA; however, considerable variation was observed with the OFF population due to the heterogeneous nature of the biofilms, and thereby, this difference was not statistically significant (Fig. 5g; *P*  = 0.062, unpaired *t* test). Multiple factors can contribute to the integration of eDNA and DNA-binding proteins into the biofilm matrix; however, lower abundance of these components is consistent with the observed decrease in overall structure of biofilms formed by the *modA2* ON population. Increased adherence by *modA2* ON bacteria, as suggested by increased bacterial density near the base of the biofilms, likely contributes to the greater overall biomass observed under alkaline conditions.

To determine if HU expression was regulated at the transcriptional level, we performed RNA sequencing to compare transcriptional profiles of NTHI within biofilms formed under alkaline conditions. RNA was isolated from 16-h biofilms formed by strain 723 *modA2* OFF and strain 723 *modA2* ON populations at 37°C at pH 9. Surprisingly, there was no significant difference in relative expression of *hupA* (HU) between *modA2* OFF and *modA2* ON populations (see Table S4 in the supplemental material). The *hupA* expression result was confirmed by quantitative reverse transcription-PCR (RT-PCR). As *hupA* transcription was not directly regulated by ModA2, alternative mechanisms, such as translational regulation or posttranslation modification may occur. Differences in eDNA abundance or composition may also affect HU integration into the biofilm matrix.

10.1128/mBio.01682-18.6 TABLE S4 NTHI strain 723 complete RNA sequencing results. Relative transcript abundance in biofilms formed by NTHI strain 723 *modA2* ON and *modA2* OFF populations at 37°C at pH 9. Differential gene expression was determined with the Bioconductor package EdgeR. Download Table S4, XLSX file, 0.2 MB. Copyright © 2018 Brockman et al. 2018 Brockman et al. This is an open-access article distributed under the terms of the Creative Commons Attribution 4.0 International license.

Based on RNA sequencing results, 9 genes were significantly upregulated and 8 genes were significantly downregulated more than 2-fold in biofilms formed by *modA2* ON bacteria compared to biofilms formed by *modA2* OFF bacteria (Table 1). The gene that encodes the high-molecular-weight (HMW) adhesin was significantly upregulated by the *modA2* ON population compared to the *modA2* OFF population during alkaline biofilm formation. Members of the HMW family of adhesins are involved in attachment to epithelial surfaces and in colonization (33, 34). Several genes that encode putative phage proteins were also overexpressed in the *modA2* ON biofilm samples. In contrast, the dimethyl sulfoxide (DMSO) reductase operon, which is involved in anaerobic respiration, was significantly upregulated in *modA2* OFF biofilms compared to *modA2* ON biofilms. Components of the nitrate and nitrite reductases were also significantly upregulated, as were several genes involved in cytochrome *c* maturation (Table S4), which suggests a shift to a more anaerobic metabolism within biofilms formed by the *modA2* OFF population.

**TABLE 1:** Significant differential gene expression in NTHI strain 723 biofilms formed by *modA2* locked ON bacteria compared to *modA2* locked OFF bacteria

Gene	Protein description	Log FC	FC	*P* value
Greater expression in *modA2* ON				
NTHI723_01859	Hypothetical protein (phage)	−2.25	4.76	1.09E−04
NTHI723_01862	Phage minor tail protein U	−1.83	3.56	1.12E−04
NTHI723_01861	Prophage minor tail protein Z (GPZ)	−1.68	3.21	3.54E−05
hxuA_1	HMW adhesin	−1.67	3.17	3.54E−05
NTHI723_01863	Hypothetical protein (phage)	−1.56	2.95	1.09E−04
*fis*	Hin recombinational enhancer-binding protein	−1.27	2.40	6.14E−03
*glxK*	Glycerate kinase	−1.22	2.33	9.08E−04
gsiA_5	Glutathione import ATP-binding protein GsiA	−1.13	2.19	2.04E−05
*clpP2*	ATP-dependent Clp protease proteolytic subunit 2	−1.10	2.15	1.09E−04
Greater expression in *modA2* OFF				
yhjE_1	Inner membrane metabolite transport protein	1.25	2.37	8.55E−03
NTHI723_00144	Hypothetical protein	1.21	2.32	4.82E−02
*dmsD*	Twin-arginine leader-binding protein DmsD	1.17	2.24	3.44E−04
*thiE*	Thiamine-phosphate synthase	1.15	2.21	3.64E−02
*ccmE*	Heme chaperone CcmE	1.09	2.14	4.25E−04
*dmsA*	DMSO reductase DmsA precursor	1.05	2.07	4.17E−04
*dmsC*	DMSO reductase anchor subunit	1.03	2.04	9.80E−05
*clpB*	Heat shock protein F84.1	1.00	2.00	1.07E−02

## DISCUSSION

In this study, we demonstrated how the phasevarions of multiple NTHI clinical isolates influenced biofilm formation under host-relevant microenvironmental conditions. The most significant differences occurred at the pH and temperature found within the ME during chronic disease. We also found that multiple phasevarions (*modA2*, -*A5*, and -*A10*), which each recognize a unique DNA sequence, regulated similar patterns of biofilm formation under specific conditions that mimic those in the ME during chronic OM (37°C, pH 9). Yet, under neutral conditions at the temperature of the nasopharynx or middle ear, biofilm formation was diverse among these strains. Furthermore, other phasevarions, such as ModA9 of NTHI strain 1209, did not affect biofilm formation under any of the conditions tested. NTHI phasevarions appeared to regulate biofilm formation in a microenvironment-dependent fashion.

Phasevarions have been identified in multiple human mucosa-associated pathogens, all of which are known to form biofilms during pathogenesis and disease. The phasevarions of several species have been shown to regulate disease-relevant phenotypes such as the ability to acquire nutrients and resistance to oxidative stress (29). Here we show for the first time that the phasevarions of multiple NTHI clinical isolates regulated biofilm formation under host-specific microenvironmental conditions, yet others, such as the ModA9 phasevarion of strain 1209, had no effect on biofilm formation. While ModA9 did not impact biofilm formation by NTHI strain 1209 under the conditions tested here, ModA9 does significantly affect other phenotypes, such as expression of the outer membrane protein OMP P6 (12). The conservation of the relationship between multiple phasevarions and biofilm formation under the conditions of chronic ME disease is remarkable and signifies the importance of phasevarion-influenced biofilm formation within this microenvironment. Laser-scanning confocal microscopy of biofilms formed at an alkaline pH revealed unique differences in biofilm architecture formed between the ON and OFF subpopulations of the *modA2* and *modA5* representative strains. Under alkaline conditions, the *modA2* ON and *modA5* ON populations formed mat-like biofilms with significantly less discrete towers and water channels than those formed by the *modA2* OFF and *modA5* OFF populations at pH 9 or biofilms formed by either subpopulation at a neutral pH. Several models of biofilm formation suggest that more heterogeneous biofilms with tower-like structures and intervening water channels may allow for better exchange of nutrients and oxygen into the biofilm, as well as removal of waste from the biofilm (35, 36). Biofilm formation and maturation are a dynamic process with ongoing changes in gene and protein expression over time (26, 37). As such, the impact of phasevarion-induced differences in biofilm architecture may become more important as the biofilms mature. Future studies that assess biofilms formed for greater than 24 h may reveal additional long-term benefits of a particular phasevarion status or switching on the persistence of NTHI within biofilms.

While the effect of changes in NTHI biofilm architecture *in vivo* is not yet known, loss of biofilm structural stability has been shown to increase bacterial dispersion in a chinchilla model of OM (38). Biofilm formation is a major factor in bacterial recalcitrance and persistence within the middle ear. Increased resistance to antimicrobials is attributed to changes in bacterial metabolism and decreased growth rates (27). Regulatory mechanisms that balance the need for nutrient acquisition and growth with the benefits of biofilm quiescence will facilitate effective microniche adaptation and survival. While NTHI biofilm formation is important in colonization of the nasopharynx, NTHI can utilize mechanisms such as the phasevarion to also regulate biofilm formation in response to stress or microenvironmental changes, such as the alkaline condition found in the ME during chronic disease.

Under alkaline conditions, the relative abundances of biofilm-associated proteins were different between biofilms formed by the *modA2* locked OFF and *modA2* locked ON populations. Differences in nutrient availability and stress could contribute to the observed changes in iron acquisition, oxidative stress, and metabolic proteins within these biofilms. In addition, differences in protein expression and biofilm formation on mucosal surfaces diminish host response and recognition and can alter the effectiveness of innate immune defenses (39–41).

Biofilms formed by *modA2* ON bacteria contained significantly less of the bacterial DNABII DNA-binding protein HU. HU and the related IHF play a critical role in biofilm structure by stabilization of extracellular DNA within the biofilm matrix (30, 42). Strategies directed against these DNA-binding proteins are highly effective at disrupting biofilms formed *in vitro* and *in vivo* and present very promising targets for biofilm prevention and treatment (38, 43). We also found that biofilms formed by *modA2* ON bacteria at an alkaline pH had relatively less eDNA than comparable biofilms formed by *modA2* OFF bacteria. Decreased extracellular DNA in biofilms formed by the *modA2* ON population corresponded with the reduced abundance of HU integrated into the adherent biomass. As eDNA and DNABII proteins are required to build and maintain the stability of the biofilm matrix, loss of these critical structural components would be expected to result in biofilms similar to the less-structured and more mat-like biofilms formed by *modA2* ON bacteria under alkaline conditions. Future work is necessary to fully understand the integration of eDNA and DNA binding proteins into the biofilm matrix under alkaline conditions and the role of the phasevarion in this process.

The most significant differences in biofilm formation occurred at an alkaline pH, and pH was the strongest driver overall for changes in the biofilm proteomes. Middle-ear effusions of children with chronic OM and chinchillas during experimental OM are alkaline in nature and can reach a pH of 9 or greater (16). Such alkaline conditions are uncommon elsewhere in the human body. The results of this study highlight the need to understand the influence of this alkaline environment on bacteria within the ME during infection. While clear differences in biofilm formation by NTHI strain 723 were obvious when grown under alkaline conditions that mimic the diseased ME, there was no difference in biofilm formation by this strain when grown under standard laboratory conditions at 37°C and at a neutral pH. Biofilm formation was affected by phasevarion status in NTHI strains 477, R2866, and C486, which carry the *modA5*, *modA10*, and *modA4* alleles, respectively. Interestingly, strain R2866, which formed biofilms with the greatest biomass and thickness under all conditions tested, is the only blood isolate assessed and also the only strain that expresses the Hia adhesin (44, 45). In strain R2866, Hia expression can be regulated via ModA10-independent phase variation of the poly(T) tract in the promoter region (12). Additional work is necessary to determine the specific role of Hia in biofilm formation by strain R2866. All other strains tested express the high-molecular-weight (HWM) adhesins. In biofilms formed at 37°C at pH 9, there was greater expression of the HWM gene by *modA2* ON compared to *modA2* OFF. Increased expression of adhesins such as HMW adhesin may contribute to greater attachment to the coverslip, and as such, the unique mat-like architecture observed with the *modA2* ON population at an alkaline pH. Regulation of the different adhesins expressed by each of these strains likely plays an important role in the biofilm phenotypes observed, as well as adherence and colonization, and will require further investigation (46).

The presence of phasevarions in pathogenic bacteria adds an extra level of complexity to understanding bacterial responses to changing conditions and microenvironments, and it is critical to understand the implication of these mechanisms for disease. Highly effective treatment and prevention strategies will require a more complete understanding of not only which virulence factors are being regulated, but also when and where they are expressed. It is also important to consider the phase of bacterial lifestyle (planktonic versus biofilm versus newly released from a biofilm) in the development of vaccines, as expression of bacterial antigens can vary significantly (47). Here, identification of genes and proteins regulated by the NTHI phasevarion during biofilm formation at disease-relevant pHs and temperatures will facilitate continued research to better understand the role of these proteins in biofilm formation over the course of disease. Furthermore, interventions that eradicate only a single *modA* subpopulation may lead to a shift in *modA* status and result in more severe disease (13). Therefore, an increased understanding of the role of the phasevarion in disease processes, such as biofilm formation, will facilitate the development of new combinatorial strategies that synergistically target the entire NTHI population regardless of *modA* status, to not only prevent but also treat chronic diseases due to nontypeable Haemophilus influenzae.

## MATERIALS AND METHODS

### Bacterial strains and culture medium.

NTHI strains 723, 477, and 1209 were received from the Finnish Otitis Media study group (48). NTHI strain C486 was isolated from a child with otitis media (49), and NTHI strain R2866 was isolated from the blood of a child with meningitis (50). NTHI cells were routinely cultured in brain heart infusion broth supplemented with hemin (2 µg/ml) and NAD (2 µg/ml) (sBHI) or on chocolate agar and grown at 37°C with 5% CO_2_.

### Biofilm formation.

Biofilms were formed by NTHI cultured within chambers of eight-well-chambered coverglass slides (Thermo Scientific, Waltham, MA) as described previously (51). Briefly, mid-log-phase cultures of NTHI strains were diluted with buffered sBHI that contained 100 mM HEPES (Fisher BioReagents), adjusted to pH 7 or 100 mM TAPS (Sigma-Aldrich), adjusted to pH 9. NTHI cells were inoculated at 4 × 10^4^ CFU in a 200-μl total volume per well and slides were incubated at 34 or 37°C, as indicated, with 5% atmospheric CO_2_. Biofilms were grown for either 16 or 24 h, with the growth medium replaced after 16 h. To visualize, biofilms were stained with LIVE/DEAD BacLight stain (Life Technologies) and fixed overnight in fixative (1.6% paraformaldehyde, 2.5% glutaraldehyde, and 4% acetic acid in 0.1 M phosphate buffer, pH 7.4). Fixative was replaced with saline before imaging with a Zeiss 510 Meta-laser scanning confocal microscope. Images were rendered with Zeiss Zen software.

### Analysis of biofilm formation and architecture.

z-stack images acquired at 63× with a Zeiss 510 Meta-laser scanning confocal microscope were analyzed by COMSTAT2 to determine biomass (μm^3^/μm^2^), average thickness (μm), roughness (*R_a_*) and percentage of area occupied by layers (52–54). Area occupied by layer was plotted as percentage of bacterial biomass coverage per 1-μm optical section from the base of the biofilm. The standard error of the mean for replicate biofilms was calculated for each individual layer with GraphPad Prism version 6.0 (GraphPad Software, San Diego, CA).

### Formation of mixed biofilms with NTHI strain 723.

Biofilms were formed as indicated above in sBHI buffered with 100 mM TAPS and adjusted to a pH of 9. NTHI were inoculated into the chambers of an eight-well-chambered cover glass as a 1:1 mixture of *modA2* locked OFF bacteria that constitutively expressed green fluorescent protein and nonfluorescent *modA2* locked ON bacteria. A total of 4 × 10^4^ CFU of NTHI (2 × 10^4^ cells of each subpopulation) were seeded in a total volume of 200 μl per well. Cultures were incubated at 37°C with 5% atmospheric CO_2_ for 24 h. The medium was replaced with fresh medium after 16 h. Biofilms were rinsed with Dulbecco’s phosphate-buffered saline (DPBS) and then counterstained with the fluorescent bacterial outer membrane stain FM4-64 (Thermo Fisher, Waltham, MA) to visualize the *modA2* locked ON subpopulation. The biofilms were fixed overnight in fixative (1.6% paraformaldehyde, 2.5% glutaraldehyde, and 4% acetic acid in 0.1 M phosphate buffer, pH 7.4) and the fixative was replaced with saline before imaging with a Zeiss 510 Meta-laser scanning confocal microscope. Images were rendered with Zeiss Zen software.

### SWATH-MS analysis.

NTHI cells were inoculated into the wells of 96-well flat-bottom microtiter plates (Corning, Corning, NY) and incubated for 16 h at 34°C at pH 7, 37°C at pH 7, or 37°C at pH 9 with 5% atmospheric CO_2_ in buffered sBHI, as described above. Biofilms were rinsed once with DPBS, and then the total adherent biomass was suspended in DPBS with aggressive scraping. The biomass was collected by centrifugation and extracted with TRIzol reagent (ThermoFisher, Waltham, MA) at 10^7^ CFU per 750 μl. Isopropanol was added to the phenol-ethanol supernatant of the TRIzol-extracted samples. Proteins were pelleted by centrifugation at 12,000 × *g* for 10 min at 4°C, washed three times with 95% ethanol, and precipitated with 100% ethanol. The protein pellets were resuspended in 200 μl 6 M guanidinium-HCl–50 mM Tris-HCl (pH 8). Proteins were then reduced and alkylated by 10 mM dithiothreitol (DTT) and 25 mM acrylamide, respectively. The resultant proteins were precipitated with 1 ml of 1:1 methanol/acetone and then suspended in 50 μl of 50 mM Tris-HCl (pH 8) with 1 μg of trypsin and incubated overnight at 37°C. Tryptic-digested peptides were cleaned with C_18_ ZipTips (Millipore, Burlington, MA). SWATH-MS analysis was performed as described previously (55).

### Detection of HU and eDNA within biofilms.

NTHI strain 723 *modA2* locked ON and *modA2* locked OFF populations that were engineered to constitutively expressed GFP (29) were inoculated into the chambers of an eight-well-chambered cover glass, as described above. After 16 h, biofilms were rinsed twice with DPBS to remove nonadherent bacteria and then incubated with anti-NTHI HU polyclonal rabbit sera (1:200) for 30 min or commercially available murine antibody against dsDNA (AbCam) (1:200) for 1 h. Biofilms were then rinsed and incubated for 15 min with goat anti-rabbit or goat anti-mouse IgG secondary antibody conjugated to Alexa Fluor 647 (Thermo Fisher, Waltham, MA). Biofilms were rinsed with DPBS and imaged with a Zeiss 700 Meta-laser scanning confocal microscope. Relative HU and eDNA abundance was calculated as total fluorescence in the Alexa Fluor 647 channel divided by total biomass in the GFP channel, multiplied by 100.

### RNA sequencing.

NTHI were inoculated into the wells of 96-well flat-bottom microtiter plates (Corning, Corning, NY) and incubated for 16 h at 37°C with 5% atmospheric CO_2_ in sBHI buffered to pH 9, as described above. Biofilms were rinsed once with DPBS, and then the total adherent biomass was collected. Total bacterial RNA was collected by acid-phenol-chloroform extraction and purified with the RNAeasy kit (Qiagen, Hilden, Germany), according to the manufacturer’s directions. Library preparation and sequencing were performed as described previously (56). Differential gene expression was determined with the Bioconductor package EdgeR (57).

### Statistical analysis.

All statistical analyses were performed with GraphPad Prism version 7.0 (GraphPad Software, San Diego, CA). The statistical tests used and *P* values are indicated within the text and figure legends. All experiments were performed a minimum of three times on separate days in duplicate.
