# Supplementary figures and images for: Epigenetic Regulation Alters Biofilm Architecture and Composition in Multiple Clinical Isolates of Nontypeable Haemophilus influenzae
Source: mBio. 2018 Sep 18;9(5):e01682-18. doi: 10.1128/mBio.01682-18 (PMC6143736; doi:10.1128/mBio.01682-18)

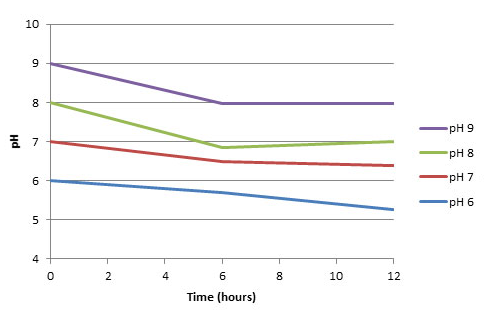

Supplement: FIG S1 [file mbo004184062sf1.tif]

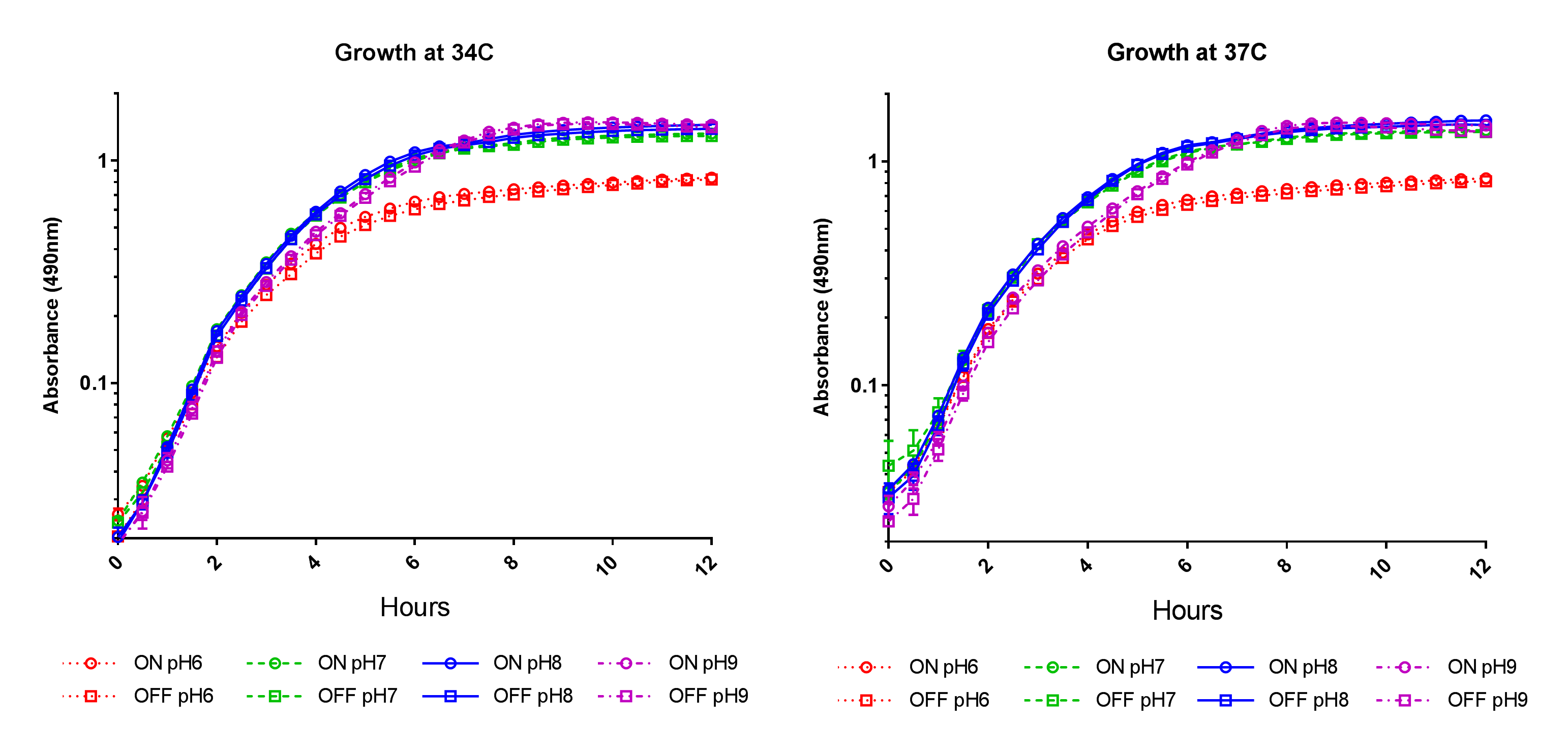

Supplement: FIG S2 [file mbo004184062sf2.tif]
